# China's model to combat the COVID-19 epidemic: a public health emergency governance approach

**DOI:** 10.1186/s41256-020-00161-4

**Published:** 2020-07-14

**Authors:** Yan Ning, Ran Ren, Gerard Nkengurutse

**Affiliations:** 1 School of Public Health, Cheeloo College of Medicine, Shandong University, Jinan, 250012 China; 2 School of Public Health, Dalian Medical University, Dalian, 116044 China; 3 Global Health Research Center, Dalian Medical University, Dalian, 116044 China; 4 International Education College, Dalian Medical University, Dalian, 116044 China

**Keywords:** COVID-19, China, Governance model, Public health emergency governance, Epidemic

## Abstract

The outbreak of Coronavirus Disease 2019 (COVID-19) is of global health concern. It is a serious public health emergency for the entire world, threatening human life and public health security. To address the epidemic, it is necessary not only to take good prevention and treatment measures, but also to have effective and targeted public health emergency governance. That said, reports focusing on governance are scant. In this commentary, we summarize China’s model to combat the COVID-19 epidemic from a public health emergency governance approach. Stemmed from goals and values, a number of mechanisms are put forward, which include: a whole-of-government response and accountability, setting up a multi-sectoral cooperation platform, swiftly scaling up epidemic emergency capacity, whole-of-society actions with engagement of social organizations, and engaging citizens in the epidemic prevention and control. As the epidemic continues to evolve, other countries might learn from China to build their own, context-specific models for better outcomes.

## Background

COVID-19 has been characterized by the World Health Organization as a public health emergency of international concern and later a pandemic. It is a serious global public health emergency threatening human life and public health security. To address the epidemic, it is necessary not only to take good prevention and treatment measures, but also to have an effective and targeted governance.

Since the epidemic outbreak to early 2020, all the 31 provincial-level regions in the Chinese mainland had initiated a first-level response. China established a nationwide joint prevention and control system to take whole-of-government and whole-of-society measures. It has taken its responsibility to safeguard the world public health security. In less than two months of the battle, China's strategy yielded positive results [1]. For the first time, China reported no domestic confirmed case on March 19^th^ [2]. As of May 31, 94.3 percent of patients had recovered (78307 discharged cases out of 83017 total confirmed cases), with a fatality rate of 5.6 percent [3]. Studies reporting governance behind these positive results can serve as experience to other countries. However, reports focusing on public health emergency governance are scant. Both China and the world need to collectively and quickly make strong emergency responses to combat the common enemy—COVID-19—as a community of shared future for mankind [4]. The objective of this commentary is to summarize, based on its practice, China’s model to combat the COVID-19 epidemic and subsequently derive policy implications.

**Fig. 1:**
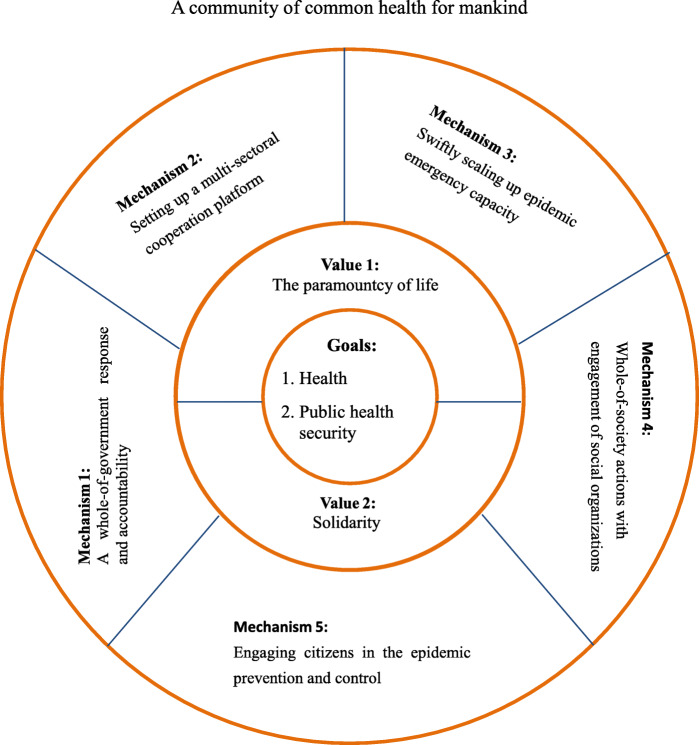
China's governance model to combat the COVID-19 epidemic

China’s model has been found to include goals, values and mechanisms (See Fig. 1). With solidarity and the paramountcy of life as core values, the main goals are to protect people’s health and to maintain public health security. Within the concept of governance for health [5], mechanisms in China’s model are those rules for solidarity to achieve engagement of all the actors (governments and other actors) in the response to the epidemic through both whole-of-government and whole-of-society approaches. They are described in details as follows.

## A whole-of-government response and accountability

In the face of a previously unknown virus and a new public health threat, a leadership system—the Epidemic Prevention and Control Headquarters System (EPCHS)—was set up at all levels, enabling a whole-of-government response and accountability to address the epidemic. It takes charge of a detailed work in its region and mobilizes broad community engagement. President Xi personally directs and deploys the work, and he chaired a number of meetings at the central level. He stated that ensuring the safety and health of the people is a top priority and major task of the government [6]. This is a sign of a strong and positive political will which is acknowledged to effect change in public health arena. COVID-19 was listed as a Class B infectious disease in the Law of the People’s Republic of China on Prevention and Treatment of Infectious Diseases, while addressing it with measures applicable to a Class A infectious disease. Under the unified commands and accountability, EPCHS runs uniformly and consistently and fulfills its duties in accordance with the law.

## Setting up a multi-sectoral cooperation platform

Multi-sectoral cooperation is an important approach to public health governance. The joint prevention and control mechanism of the state council (JPCMSC), which consists of 32 sectors, was set up on January 21^st^, 2020 as a new coordination platform of multiple ministries at the central people's government level [7]. They have clear responsibilities and divisions, including but not limited to epidemic prevention and control, scientific research, publicity, foreign affairs and logistics support. The JPCMSC has played a crucial role in organizing collective actions against the epidemic and coordinated the multi-sectoral cooperation. It gives a joint news release conference on a daily basis on China Central Television (CCTV) and ensures transparency about the epidemic facts to the public.

## Swiftly scaling up epidemic emergency capacity

In response to the dramatic increase in cases and the shortage of health resources, the National Health Commission (NHC) fleetly mobilized 42,600 multidisciplinary medical workers and 965 public health workers along with large amounts of technical equipments internally from other provinces to support Hubei (the province where resides Wuhan, the former epicenter). Particularly, over 11,000 doctors and nurses with expertise in critical care (almost 10 percent of China's critical care workers) were sent to Wuhan [8]. Cabin hospitals—transformed from public venues such as exhibition centers and gymnasiums in Wuhan—accommodated thousands of patients. Moreover, two makeshift hospitals (Lei Shenshan and Huo Shenshan hospital) were built within a couple of days. They enabled the health system to meet the urgent health needs— to test, isolate and treat every case. This shows that mechanisms that allow challenging and time-sensitive decisions to allocate limited resources should be available.

## Whole-of-society actions with engagement of social organizations

Diverse social organizations (such as foundations, enterprises and volunteers) participate in the epidemic control through various ways with a strong sense of corporate social responsibility. Their engagement makes the public health emergency response more efficient and effective. They organize plenty of donations (such as personal protective equipment (PPE) and other medical equipments) to support those in urgent need, share expertise and provide several social services. For instance, Alibaba Group has given a full play to its advantages as a digital platform enterprise and mobilized a large amount of resources to help in the global fight against the pandemic through various actions [9]. Alipay also launched a unified national prevention and control information code (“Health Code”). The Health Code is a useful assistant to facilitate citizens’ movement and to underpin local specific containment measures.

## Engaging citizens in the epidemic prevention and control

Governance needs operationalization by individuals at lower levels. Citizens' engagement allows self-management and active participation in the epidemic prevention and control at the individual level. Mass media fostered population involvement and active participation in the battle, exemplifying what e-governance may offer in the future [5]. It also improved personal public health literacy and skills through disseminating health education (e.g. washing hands). As a frontline defense in the neighborhood, community staffs and volunteers broadcast COVID-19-related knowledge and prevention measures to the residents. They also provide necessary daily services such as purchasing food. These efforts increase availability of information and social support in solidarity. Residents have a sense of responsibility to comply with social distancing measures, such as staying at home, closing schools, shutting businesses and entertainment. Particularly the Wuhan citizens showed a good compliance to Wuhan’s announcement of a citywide lockdown on January 23^rd^. This incarnates attainment of a shared goal between citizens and the government—Health and combatting COVID-19 as a top priority. Without the aggressive action, there would have been over 700 thousand of confirmed cases outside Wuhan by 19 February, day 50 of the epidemic [10].

## Policy implications

China’s experience has been proved to be effective. However, lessons from several public health interventions show that success in one country may not necessarily imply success in another due to social, economic, political and cultural factors. Therefore, other countries shall learn from China based on their own national context and global experience. In particular, a number of policy implications can be derived from China’s governance model:

1. Making people’s lives and health as a top priority

The basic tenet is to save lives and protect people’s health by stopping the epidemic, based on the paramountcy of life. China adopted extensive, stringent measures to cut the transmission channels of the virus in order to achieve these goals. It made an all-out effort to test, isolate and treat every case with the ultimate goals of increasing detection and cure rates while lowering infection and fatality rates.

(2) Emphasizing whole-of-government and whole-of-society actions

Government, social organizations and citizens all engaged in combatting the epidemic in China. Every actor showed a strong sense of responsibility by participating in collective actions. Moreover, the joint platform ensured multisectoral coordination and cooperation. Several resources and innovative technologies in the whole nation were rapidly mobilized and reallocated to combatting the epidemic.

(3) Working towards building together a community of common health for mankind

This virus has no borders and countries are interconnected and interdependent. Therefore, only by working together can countries guarantee success. Solidarity is the basic principle and center of all efforts to defeat COVID-19 and promote resilience.

## Data Availability

Not applicable.

## Abbreviations

COVID-19: Coronavirus Disease 2019
EPCHS: Epidemic Prevention and Control Headquarters System
JPCMSC: The joint prevention and control mechanism of the state council
CCTV: China Central Television
NHC: The National Health Commission
PPE: Personal protective equipment
